# A Case of Neonatal Pyriform Sinus Cyst Presenting With Respiratory Distress

**DOI:** 10.7759/cureus.84878

**Published:** 2025-05-27

**Authors:** Nayu Yokoyama, Manabu Komori

**Affiliations:** 1 Department of Otolaryngology, St. Marianna University School of Medicine, Kawasaki, JPN

**Keywords:** branchial pouch, cervical cyst, congenital neck mass, ex utero intrapartum treatment, laryngoscopy, neonatal airway obstruction

## Abstract

Pyriform sinus cysts are rare congenital anomalies originating from the third or fourth branchial pouch that can cause neonatal airway obstruction. We report the case of a male neonate born at 38 weeks of gestation who developed respiratory distress at birth. Physical exam revealed subcostal retractions and nasal flaring, but no stridor. Laryngoscopy on day 4 demonstrated a bulging posterior pharyngeal wall, and computed tomography (CT) identified an air-filled cyst extending into the left neck, suggestive of a pyriform sinus cyst. Ultrasound (US)-guided aspiration of 7 mL of fluid temporarily relieved symptoms; however, recurrence necessitated surgical excision on the seventh day of life. The cyst and two fibrous cords were removed. Histology analysis confirmed infection with chronic inflammation and granulation tissue. The patient developed transient left vocal cord paralysis, which resolved within three months. Follow-up magnetic resonance imaging (MRI) showed no recurrence. This case highlights the importance of early diagnosis and surgical intervention to prevent airway obstruction, while emphasizing the roles of imaging, nerve preservation, and postoperative monitoring. Further research into minimally invasive techniques and prenatal diagnosis is warranted.

## Introduction

Pyriform sinus cysts and fistulas are congenital anomalies resulting from incomplete involution of the third or fourth branchial pouch during embryogenesis [1,2]. Clinically, they are classified under the broader category of branchial cleft anomalies. Remnants of the first and second pouches are located in the parotid or upper cervical region, whereas derivatives of the third and fourth pouches extend deep to the thyroid cartilage and open at the apex of the piriform sinus. Cystic dilatation or incomplete involution of this tract leads to the formation of a pyriform sinus cyst or fistula [3,4]. Most cases are diagnosed during infancy or later childhood, often presenting as recurrent thyroiditis or a lateral neck mass [5]. However, when they present in the neonatal period, they may cause life-threatening airway obstruction.

Early diagnosis in neonates is challenging, and delays in recognition can worsen the prognosis. Ultrasound (US) is recommended as a first-line imaging modality and can detect abnormalities associated with a pyriform sinus fistula as early as 18 weeks of gestation on prenatal scans [5]. Endoscopy, computed tomography (CT), and magnetic resonance imaging (MRI) also provide valuable diagnostic information [6]. Anatomically, the laryngeal inlet forms the anterior wall of the pyriform sinuses, which are the lateral recesses of the hypopharynx. The persistent fistulous branchial pouch-derived tract typically courses posterior to the thyroid cartilage and terminates near the upper pole of the thyroid gland, running in close proximity to the recurrent laryngeal nerve [3,4]. Aspiration or biopsy of cyst contents may offer additional diagnostic confirmation. Definitive management involves complete surgical excision of the cyst along with any associated fistulous tract [7]. Because the tract lies adjacent to the recurrent laryngeal nerve, neonatal surgery carries a risk of transient or permanent vocal cord palsy and therefore requires meticulous operative planning and vigilant postoperative monitoring.

We present the case of a neonatal pyriform sinus cyst diagnosed following early-onset respiratory distress. This report provides a detailed account of the diagnostic process, treatment strategy, and clinical challenges unique to the neonatal population.

## Case presentation

The patient was a male neonate born at 38 weeks and 2 days of gestation via uncomplicated vaginal delivery, with a birth weight of 3,145 g. Apgar scores were 7 at one minute and 9 at five minutes. There was no evidence of meconium-stained amniotic fluid. Immediately after birth, the infant showed weak crying and required airway suctioning and continuous positive airway pressure (CPAP). By 1.5 hours after delivery, his respiratory status had improved sufficiently to discontinue positive pressure ventilation. However, persistent subcostal retractions and grunting necessitated transfer to a neonatal intensive care unit (NICU).

Upon admission to our NICU on day 1 of birth, the patient’s vital signs were as follows: body temperature 37.3°C, blood pressure 62/33 mmHg, heart rate 130 breaths per minute (bpm), respiratory rate 60 bpm, and oxygen saturation (SpO₂) ranging from 88% to 96% on room air. Physical examination revealed good activity and spontaneous crying; however, subcostal retractions and flaring were continuously present. No inspiratory stridor was noted during crying, and there were no cardiac murmurs or visible external malformations. There was no hoarseness in the voice. 

Laryngoscopy performed on day 4 revealed bulging of the posterior pharyngeal wall. No vocal cord paralysis or laryngeal malformations, such as laryngomalacia, were observed. Contrast-enhanced CT identified an air-containing cystic lesion extending from the posterior pharyngeal wall to the left side of the neck. Based on these findings, a pyriform sinus cyst was suspected. As no palpable cystic mass was detected, US-guided percutaneous needle drainage was performed. Seven milliliters of serous fluid was aspirated, resulting in temporary improvement of respiratory symptoms. Cytological analysis of the fluid revealed the presence of *Escherichia coli*. Although the patient was afebrile and showed no other signs of infection, the culture findings suggested infection; therefore, broad-spectrum antibiotic therapy with ampicillin and sulbactam was initiated.

Follow-up with US performed the next day revealed cyst recurrence, measuring 0.5 cm in diameter, along with fluid reaccumulation. Attempts to re-aspirate the cyst were unsuccessful due to the softness of the cyst wall, which made puncture difficult. Moreover, the procedure was aborted due to desaturation caused by airway compression. Although oxygen saturation normalized promptly after discontinuing the procedure, surgical intervention was deemed necessary. However, as the patient was stable, immediate surgery was not performed, and the procedure was scheduled for 10 days later. However, on the following day, the second day following the initial aspiration, the mass had enlarged further, raising concern for significant airway compression. To secure the airway, the neonatologist performed tracheal intubation at the bedside, and the surgery was rescheduled to take place seven days earlier. Intubation was uncomplicated, and no special procedures or complications occurred. 

**Figure 1:**
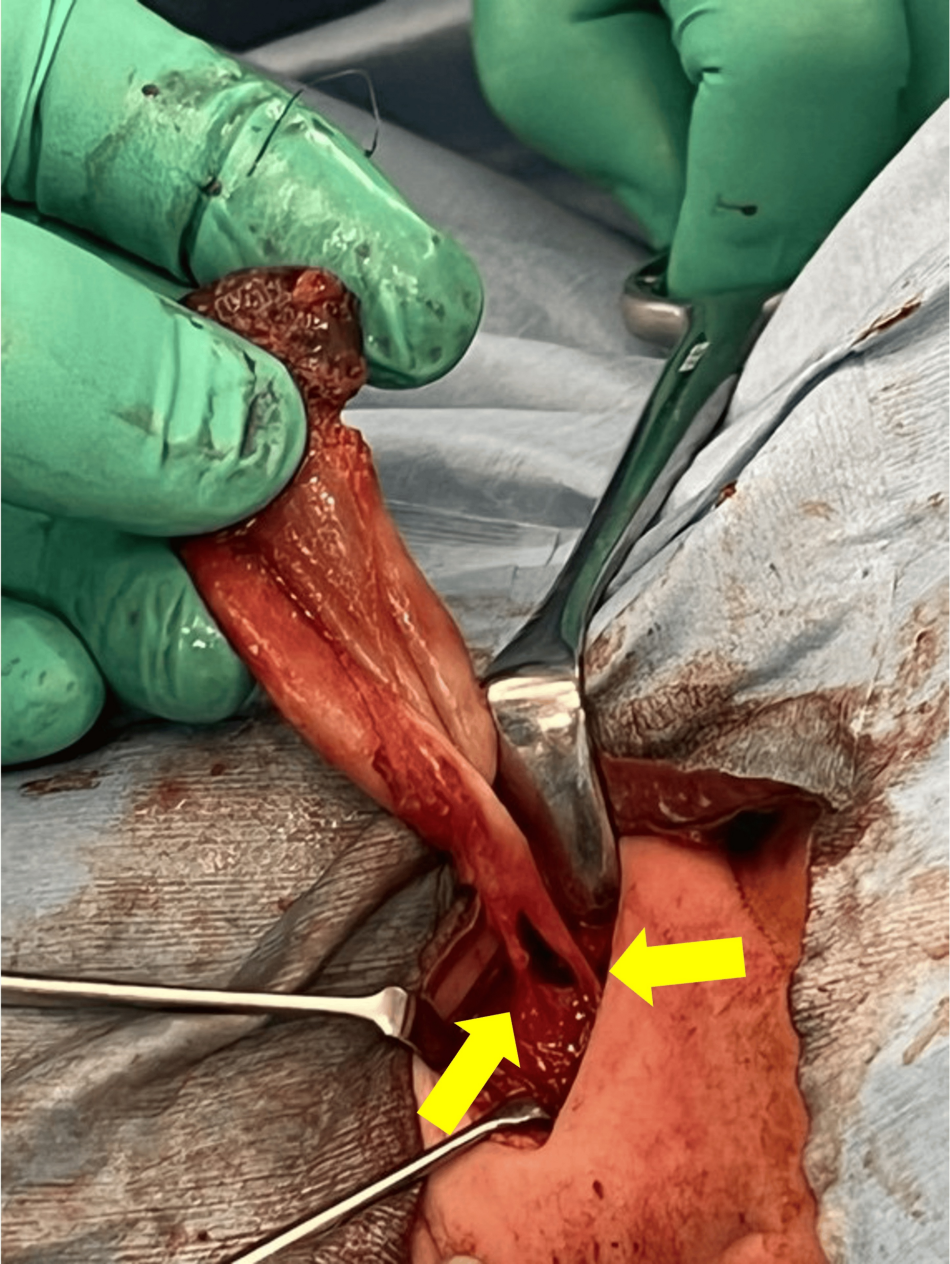
Removal of the pyriform sinus cyst Arrows indicate two associated cord-like structures.

Surgery was performed via an external cervical incision to expose the pyriform sinus cyst, followed by circumferential dissection along the cyst wall. The sternocleidomastoid muscle, which was displaced laterally by the mass, was largely preserved, while the medial strap muscles were divided. The internal jugular vein and common carotid artery were identified posterolateral to the lesion. Dense adhesions were released from the tracheal wall. Two cord-like structures in continuity with the cyst were ligated and divided before the specimen was removed completely (Figure 1). The recurrent laryngeal nerve was identified and carefully preserved. Intraoperative nerve monitoring could not be employed, as there are no pediatric endotracheal tubes compatible with nerve monitoring.

Before surgery, an attempt was made to identify the fistula via transoral direct laryngoscopy and pyoktanin blue staining. However, due to the large size of the cyst, the fistula could not be visualized. Consequently, continuity between the cyst and the cord-like structure could not be confirmed, yet their bases were ligated during surgery to prevent recurrence. Intraoperative preservation of the recurrent laryngeal nerve was successfully confirmed visually. Histopathological examination of the excised specimen was consistent with purulent infection (see "Pathological findings" section).

Extubation was performed on postoperative day (POD) 3, after stabilization of the infant’s respiratory status. Immediately following extubation, the patient developed suprasternal retractions and marked hoarseness. Laryngoscopy demonstrated left vocal cord paralysis, which was not observed prior to surgery. High-flow nasal cannula (HFNC) support and intermittent nebulized adrenaline were started. The retractions subsided after the first dose. Adrenaline was discontinued on POD 6, and HFNC was weaned by POD 8. Since postoperative recurrent laryngeal nerve palsy in neonates is typically transient and resolves without specific intervention, no further medical or surgical treatment was provided in this case [8]. The infant received nutrition via a nasogastric tube up to POD 8 and began oral feeds on POD 9. The patient was managed expectantly with close monitoring. By POD 34, the hoarseness had resolved, and at the three-month follow-up, vocal cord mobility had returned to normal with no MRI evidence of cyst recurrence.

## Discussion

Pyriform sinus cysts are congenital anomalies originating from the third or fourth branchial pouch [1,2], which can form cysts or fistulas in the airway or neck [3]. These lesions are typically diagnosed in infancy or later in childhood, often presenting as recurrent subacute thyroiditis or cervical masses [7]. Diagnosis during infancy can be complicated by recurrent infections and prior treatment history. In contrast, pyriform sinus cysts in the neonatal period pose additional diagnostic challenges and require urgent recognition, as they may lead to airway obstruction and severe respiratory distress, as seen in the present case. This condition can be life-threatening, highlighting the need for prompt diagnostic and therapeutic intervention to avoid serious complications.

Diagnosis 

Neonatal pyriform sinus cysts are difficult to diagnose due to their rarity and nonspecific early clinical presentation. The main diagnostic approaches include endoscopic examination and advanced imaging techniques. US is recommended as a first-line imaging modality and can detect abnormalities associated with a pyriform sinus fistula as early as 18 weeks of gestation on prenatal scans. In the present case, routine second- and third-trimester US scans performed at the referring obstetric clinic were reported as normal, with no fetal neck mass or airway abnormality identified. The original images were not available for review after referral.

**Figure 2:**
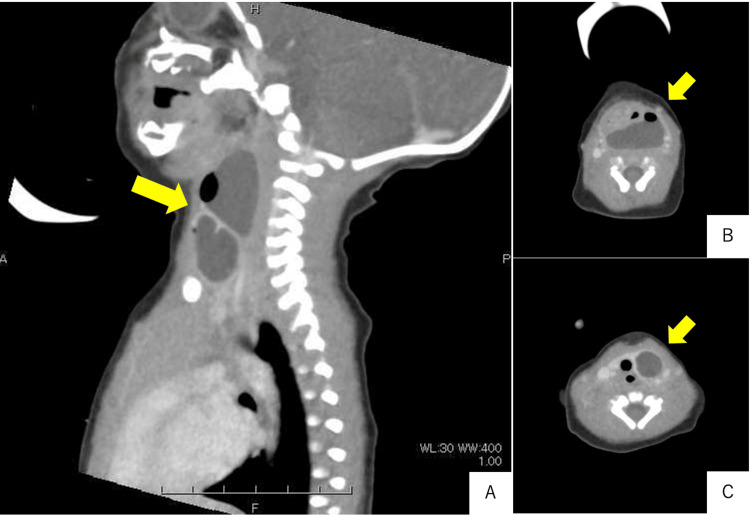
Contrast-enhanced CT indicating an air-containing cystic lesion extending from the pharynx to the neck (arrows) (A) Sagittal plane. (B) Axial plane, C4 level. (C) Axial plane, C6 level.

Bedside laryngoscopy was first performed to exclude supraglottic obstruction and to assess the need for emergent airway control. It revealed bulging of the posterior pharyngeal wall. Contrast-enhanced CT was then selected since it can be acquired rapidly under brief sedation and is superior to MRI in demonstrating air fluid levels. It identified an air-containing cystic lesion extending from the pharynx to the neck (Figure 2). 

The presence of intralesional air strongly favors a pyriform sinus cyst or fistula and helps distinguish it from lymphatic malformations, vascular malformations, and thyroglossal duct cysts, which are typically fluid-filled and lack gas bubbles [8,9]. MRI was reserved for postoperative follow-up, where its excellent soft tissue contrast allows detection of residual or recurrent cysts without additional radiation exposure. 

In addition, aspiration cytology and swallowing studies, including direct laryngoscopy, can provide supplementary diagnostic information. In the present case, US-guided aspiration yielded serous-appearing fluid. However, cytology demonstrated gram-negative bacilli with *E. coli* growing in culture, confirming that the cyst was already infected despite its clear gross appearance. Endoscopic identification of fistulous openings can further improve diagnostic accuracy, although continuity of the fistula tract was not fully confirmed intraoperatively in this patient.

Treatment 

The management of pyriform sinus cysts typically involves a two-step approach: initial symptom relief by drainage, followed by definitive surgical excision. Infected cysts, as in the present case, require prompt aspiration and antibiotic therapy to control acute inflammation and reduce systemic risk.

Definitive treatment typically involves open surgical excision, which removes both the cyst and fistula and minimizes recurrence [7]. Because the tract frequently lies adjacent to the recurrent laryngeal nerve, the operation carries a risk of transient or permanent vocal cord paralysis [3,10]. In the present case, recurrence after initial drainage necessitated complete resection. Two cord-like structures in continuity with the cyst, interpreted as fistulous tracts, one coursing toward the esophagus and the other posterior to it (Figure 1), were doubly ligated and divided. No recurrence has been observed during follow-up. A transient left vocal cord paresis appeared postoperatively but resolved within three months, underscoring the importance of meticulous nerve identification and postoperative surveillance.

Minimally invasive alternatives, such as endoscopic cauterization, have been increasingly reported in older children and adults [11]. These approaches offer shorter recovery time and lower surgical risk but may fail to achieve complete fistula closure, especially in cases with large cysts or active inflammation. In neonates with severe airway obstruction, open surgery remains the first-line treatment. Given the potential severity of the condition, early surgical intervention within the first seven days of life is ideally recommended [3].

In neonates, leakage of orally administered formula through the tract into the esophagus is a well-recognized source of infection. Zhu et al. reported infection in 57.9% of neonatal pyriform sinus cyst cases overall, including 13.3% occurring within the first seven days of life [8]. Another report documented resolution of inflammation, and eventual closure of a recurrent tract, after switching from oral feeds to gastric tube nutrition [8]. In our patient, no oral feeding occurred until POD 9, yet purulent material was present by day 4. We therefore presume that bacterial invasion occurred either in utero, via aspiration of contaminated amniotic fluid through the patent tract, or during delivery, followed by bacterial overgrowth within the cyst.

Pathological findings 

Histopathological examination plays a critical role in confirming the diagnosis and elucidating the biological behavior of the lesion. Typical findings in pyriform sinus cysts include fibrous cyst walls lined by pseudostratified squamous or ciliated columnar epithelium, accompanied by chronic inflammatory cell infiltration and granulation tissue [4].

**Figure 3:**
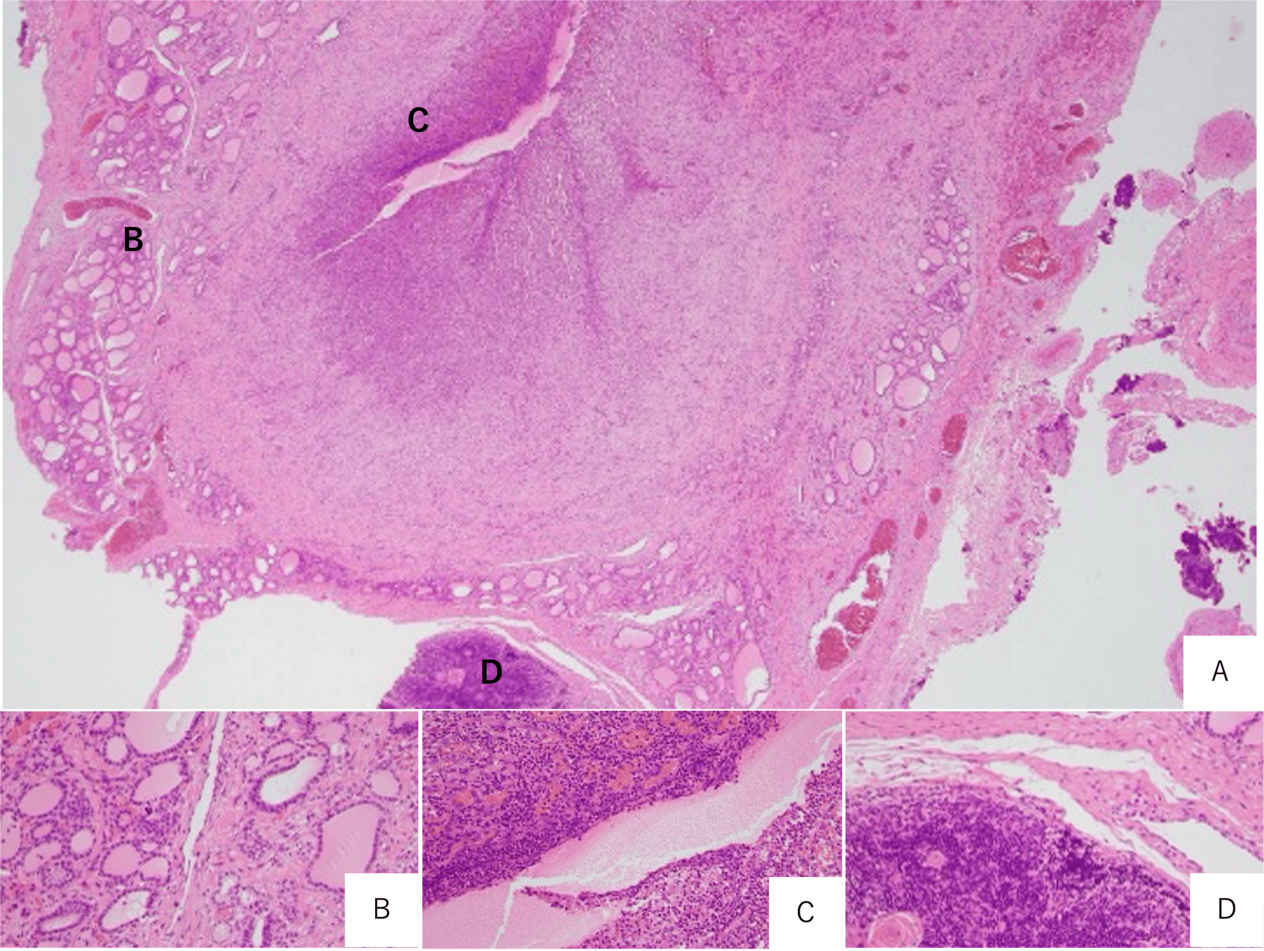
H&E staining (A) Fistula-like structure containing purulent and fibrinous exudate, along with granulation, fibrosis, and inflammatory tissue, extends and involves both the thymus and thyroid glands. (B) Cyst containing purulent material. (C) Pseudostratified squamous epithelium accompanied by chronic inflammatory cell infiltration. (D) Granulation tissue.

In the present case, histopathological examination of the specimen submitted intraoperatively demonstrated abundant purulent exudate containing neutrophils and fibrin, together with dense neutrophilic infiltration of the cyst wall and surrounding granulation tissue, using hematoxylin-eosin (H&E) staining (Figure 3). These findings, combined with the growth of *E. coli* from the initial aspirated fluid, are consistent with an active, purulent infection of the cyst at the time of surgery.

The presence of chronic inflammation underscores the importance of early and complete surgical intervention.

Clinical implications 

Neonatal pyriform sinus cysts are rare yet potentially life-threatening congenital anomalies. This case highlights the critical importance of rapid diagnosis using endoscopic and imaging modalities, followed by timely surgical excision. Furthermore, it documents two fistulous cords emanating from a single cyst, an anatomic configuration reported in fewer than 5% of neonates in the largest published series [8], and demonstrates that such dual tracts can be ligated en bloc without recurrence. Another notable aspect of this case is that infection was already evident by the fourth postnatal day, despite aspiration of serous fluid and withholding of oral feeding until POD 9. This observation supports the hypothesis that bacterial colonization may occur antenatally or during delivery rather than via postnatal reflux associated with feeding. Such findings expand the recognized clinical spectrum and highlight the diagnostic significance of identifying an air fluid level on early CT imaging, which facilitated differentiation of the lesion from lymphatic or vascular malformations.

Surgical management of neonatal pyriform sinus cysts remains challenging, particularly due to the risks of nerve injury and recurrence. Future advancements in diagnostic protocols, including prenatal imaging and fetal intervention such as ex utero intrapartum treatment (EXIT), may further improve outcomes with airway-compromising lesions [12]. These challenges underscore the need for continued refinement of surgical and minimally invasive techniques. Moreover, the integration of molecular and histopathological research may provide deeper insights into the pathogenesis of these lesions and facilitate the development of novel therapeutic strategies.

## Conclusions

This report underscores the importance of early diagnosis and prompt treatment in a case of neonatal pyriform sinus cyst presenting with respiratory distress. The active use of laryngoscopy and imaging techniques, in combination with timely surgical intervention, contributed to a favorable clinical outcome. Continued efforts to enhance diagnostic and therapeutic strategies for neonatal airway and cervical lesions remain essential. 
